# Cycle Performance of Aerated Lightweight Concrete Windowed and Windowless Wall Panel from the Perspective of Lightweight Deep Learning

**DOI:** 10.1155/2022/3968607

**Published:** 2022-06-03

**Authors:** Xing Yuan, Yao Zhang, Qinggang Lu, Shuhang Zhang, Hua Liu, Mingchang Jin, Feng Xu

**Affiliations:** ^1^College of Civil Engineering, Nanjing Tech University, Nanjing 211816, China; ^2^Beijing Institute of Architectural Design, Beijing 100045, China; ^3^Tianjin Architecture Appraisal & Design Institute, Tianjin 300381, China

## Abstract

This paper aims to explore the seismic mechanical properties of newly developed fabricated aerated lightweight concrete (ALC) wall panels to clarify the interaction mechanism between wall panels and structures. It first introduces the lightweight deep learning object detection algorithm and constructs a network model with faster operation speed based on the convolutional neural network. Secondly, combined with the deep learning object detection algorithm, the quasi-static loading system is adopted to conduct the repeated loading test on two fabricated ALC wall panels. Finally, the hysteresis load-displacement curve of each test is recorded. The experimental results show that the proposed deep learning algorithm greatly improves the operation speed and compresses the model size without reducing the accuracy. The lightweight deep learning algorithm is applied to the study of the slip performance of the wall plate. The pretightening force of the connecting screw characterizes the slip performance between the wall plate and the structural beam, thereby affecting the deformation response of the wall plate when the interstory displacement increases. The hysteresis curve of the ALC wall panel has obvious squeezing effect, indicating that the slip of the connector can unload part of the external load and delay the damage of the wall panel. The skeleton curve suggests that the fabricated windowless ALC wall panel has higher positive and negative initial stiffness and bearing capacity than the fabricated windowed wall panel. However, the degradation analysis of the stiffness curve reveals that the lateral stiffness deviation of the fabricated windowless ALC wall panel is more obvious. It confirms that the proposed connection method based on the lightweight deep learning model can improve the seismic performance of ALC wall panels and provide reference for the structural analysis of embedding fabricated ALC wall panels. This work shows the important practical value for exploring the application effect of embedded ALC wall panels.

## 1. Introduction

With the acceleration of urbanization, the urban population is increasing sharply. Improvements to the quality of life and the living environment of residents are a key concern in social development. Hence, the urban construction industry in China is developing rapidly, and the construction market is booming [1]. On the other hand, it is a basic task to explore a building system and development strategy, which are suitable for the modernization of the housing industry. In actual engineering practice, steel structure houses are widely applied because of their flexible layout, short production cycle, lightweight, good performance, and strong seismic performance. The main structural components of traditional steel structures have basically realized factory prefabrication and on-site installation, but the building envelope system still mostly uses traditional masonry walls, which require a number of wet work procedures such as masonry and on-site plastering. Due to the high cost of time and labor, it is not conducive to further improving construction efficiency and construction quality [2]. To this end, some researchers and designers have developed a series of lightweight composite wall panels, including prefabricated lightweight aggregate concrete composite wall panels, aerated lightweight concrete (ALC) wall panels, ceramsite concrete sandwich panels, foamed cement composite wall panels, and composite keel insulation wall panels [3, 4]. Among them, ALC wall panels are the most widely used.

The structural properties of ALC wall panels in practical engineering are closely related to the material properties of ALC materials and the performance of the connecting joints between the wall panels and the main structure. ALC wall panels are widely used in building at this stage, which refers to high-performance autoclaved lightweight aerated concrete panels, which are made of cement, lime, and sand [5]. The mechanical properties of ALC wall panels before bending and cracking are basically the same as those of ordinary concrete panels, but their overall stiffness and strength are lower [6]. Studies have shown that increasing the density can greatly improve various mechanical properties of wall panels but has no significant effect on improving bending. The flexural, shear, and crack resistance of ALC wall panels increase with the increase of reinforcement ratio [7]. In addition, the failure of the wall panel goes through three stages with the increase in reinforcement ratio, including bending failure, bending shear failure, and shear failure [8, 9]. The overall mechanical properties of ALC wall panels meet the requirements of building envelope panels and are suitable for various concrete and steel structures [10]. The connection methods of ALC wall panels and the main structure can be divided into inclined rod connection, bolt connection, and swing connection. Among them, the out-of-plane strength of the oblique rod connection is relatively low and the brittleness is relatively large [11]. The out-of-plane strength of the swing joint is high, and the failure is mainly the yield of the connector. The failure modes of bolted joints can be divided into three types: wall plate punching failure, wall plate delamination, and bolt shank fracture [12]. For bolted joints, the joints have excellent performance in magnitude 6 earthquakes, and the wall panels will be damaged to varying degrees under the action of earthquake accelerations above magnitude 6. Installing dense steel mesh at the joint can effectively improve the out-of-plane bearing capacity of the wallboard [13]. There is a specific synergy between the external ALC wall panels and the main structure [14]. The ALC wall panels in this work are still concrete wall panels. In practice, the shape of the wall panel changes, which affects the mechanical properties of the wall panel.

During the development of deep learning technology, the depth of the model has been continuously deepened and the amount of computation and parameters of the model are also increasing simultaneously. In recent years, researchers have paid more attention to the efficiency of deep learning models. At present, the common methods to improve the efficiency of deep learning models include pruning, compression, and quantization [15]. Lightweight convolutional neural networks (CNNs) such as MobileNet, ShuffleNet, and EfficientNet greatly reduce the amount of computation and parameters. In this work, the lightweight target detection model is improved through the research and analysis of the lightweight deep learning model and a target detection algorithm with better performance is proposed and applied in the research of wall and steel frame. The materials and methods are first introduced, including experimental materials and test plans, and then, the lightweight deep learning target detection algorithm is improved and the ALC wall panel's connection joints are designed. Finally, the lightweight deep learning algorithm and the performance of the wall panels are tested. This work innovatively connects the ACL wall with the steel frame structure and obtains their ultimate bearing capacity through experimental analysis, so as to analyze the overall seismic performance. This study aims to evaluate the seismic structural performance of the assembled ALC wall panels under different interstory deformations of the structure. Therefore, cyclic investigations were performed on the assembled ALC wall panels of the two cases (with window and without window) with designed connection joints. The test results show that the wall panels exhibit an excellent structural performance under imposed cyclic loading and the slipping behavior of connection joints remarkably impacts the performance of wall panels and could postpone the damage of the wall panels. This study could provide scientific and effective reference materials for subsequent related research in the application analysis of wall panels in practical cases. The data in this work are obtained according to their own conditions. During the experiment, the DH3826 dynamic strain collector is used to automatically collect and record the load data. The lightweight deep learning target detection algorithm uses the MS COCO (Microsoft Common Objects in Context) dataset. The hysteresis curve of ALC wall panels and the results of bolt preload between the ALC wall panels and the bolts can delay the damage of the connection can be obtained.

## 2. Materials and Methods

### 2.1. Experimental Materials

Research on the design of ALC wall panels is particularly extensive. The optimal materials and mass production of ALC wall panels have greatly promoted the technological innovation of ALC wall panels [16, 17]. The main research objects of this study are assembled ALC wall panels and series of connecting components developed by Beijing Building Materials Group (BBMG) and Beijing Institute of Architectural Design (BIAD). By adopting cross-penetrating steel bar tension and pretensioning to connect multiple ALC wall panels, the concrete monolithic wall forms a whole, which shows the characteristics of good integrity and of integrated installation and construction. Effective and reliable theoretical analysis and experimental verification are performed targeting the basic application of ACL wall panels, aiming to promote the actual engineering application of the product.

Based on the current research studies of theoretical analysis and application experience summary, it can be seen that the ALC wall panels still have the following shortcomings, which need to be verified in the practical application of the building structure. Firstly, the interaction mechanism among the ALC wall panels under repeated low-cycle loads is still unclear [18]. Secondly, the lateral stiffness, lateral bearing capacity, energy dissipation performance, ductility, and failure mode of the ALC wall panels have to be further verified [19]. Based on the experimental tests, the cycle tests are performed for the target-assembled ALC wall panel under horizontal low-cycle repeated loading in this study to examine their cyclic performance. The mechanical characteristics and failure mechanism of ALC wall panel under repeated low-cycle loading are analyzed to explore the interaction mechanism among the ALC wall panels and the characteristics of cracks among the panels. The performance of the ALC wall panels is analyzed comprehensively based on the load-displacement hysteresis curve, the overall wall panel bearing capacity, the skeleton curve, and the stiffness degradation.

### 2.2. Experimental Scheme

The Efficient Det series can perform target detection by taking Efficient Net as the feature extraction network and undertaking the bidirectional feature pyramid network as the multiscale feature fusion method. Similar to Efficient Net, Efficient Det has fewer parameters and computations compared to one-stage object detection models with similar accuracy. Based on the lightweight target detection model Efficient Det-D0, the inference delay and memory usage of detection are improved, which is called MEfficient Det (More Efficient Detector). Efficient Det uses the Swish activation function, which has the characteristics of smoothness and derivation and has good performance on deep models, but its disadvantage is that it includes two multiplications and one exponential operation, which is time-consuming for the computer. In order to pursue lower inference delay, MEfficient Det uses the Leaky ReLU (Rectified Linear Unit) activation function in the shallow feature extraction network part compared to Efficient Det-D0 and adopts the Hard-Swish activation function in the deep model, that is, the feature fusion and detection network. The Leaky ReLU activation function only includes one multiplication operation, the Hard-Swish activation function includes two multiplication and division operations, and the operation time is much lower than that of Swish. But the functional properties and function curves of the three are similar, and the actual performance in classification and target tasks is only slightly worse than the latter.

To maximize the detection rate and accuracy, MEfficient Det adjusts the structure of MBConv by analyzing the basic unit MBConv of Efficient Det. First, the SE (Squeeze-and-Excitation) module introduces a pooling operation and two convolution kernel 1 × 1 convolution operations. A large number of element-level operations will seriously affect the running rate. SE automatically learns the importance weight of each channel feature by integrating the information of each channel through average pooling and integrates the global information by multiplying the weight and the feature map element by element on the channel dimension. To enhance or suppress the feature information of each channel, the learned effective feature map channels are enhanced and the useless feature map channels are suppressed. In fact, in addition to the semantic features of the target, the video target detection task needs to retain the spatial location information as much as possible, so that the network model can obtain better localization performance. For targets with little feature information, such as small targets, and the location information passed layer by layer, SE will further weaken their feature information, which in turn leads to a decrease in the performance of the detection model. Secondly, the biggest disadvantage of lightweight networks is that the accuracy rate is lower than that of larger-scale models. CSP (Cross Stage Partial) is an efficient method to enhance the learning ability of the network. CSP adopts the cross-stage connection, that is, spanning the parts with the same input and output dimensions, and recombines the feature maps of one part of the input through the same MBConv repetition stage and the other part without the MBConv in a cascaded manner. Different channels can obtain richer gradient fusion information and enhance the learning ability of lightweight CNNs. The performance research of wall panels in the following sections is carried out in the context of lightweight deep learning algorithms.

**Figure 1:**
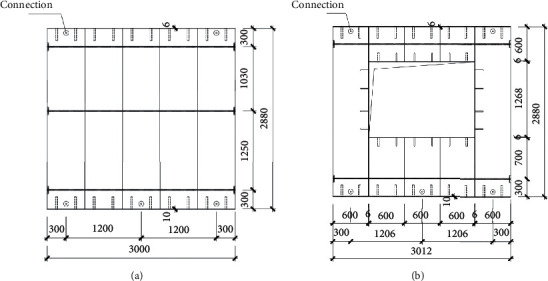
The structural design diagram of the embedded ALC integrated wall panel. (a) The wall panel without windows. (b) The wall panel with windows.

**Figure 2:**
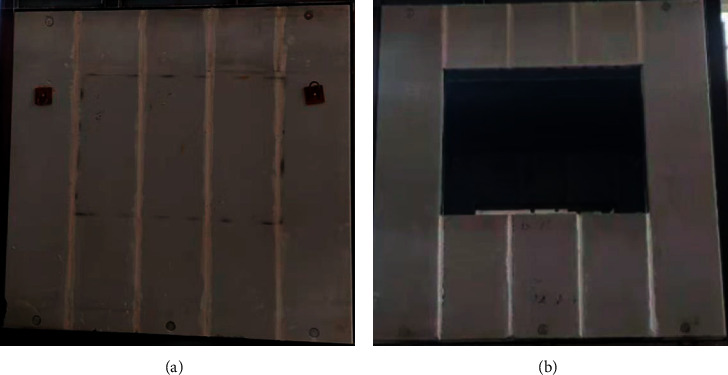
The physical diagram of the embedded ALC integrated wall panel. (a) The wall panel without windows. (b) The wall panel with windows.

The performance of the assembled ALC wall panel is tested under low-cycle repeated loading. In the present test research, two types of assembled ALC wall panels were utilized, including the unit wall without and with windows opening, denoted as Type a and Type b, which are, respectively, illustrated in Figure 1. The two types of assembled ALC wall panels both contain 5 pieces of ALC strip panels. For Type a wall panel, three tendons are arranged along the height and prestressed to assemble the strip panels as a wall unit. In addition, the 6 mm thick steel plates are anchored by L-shape angle steel at both ends of the strip panels. For Type b wall panel with window opening, besides the two prestressed tendons and steel plates used to assemble the AAC strip panels, the 6 mm thick steel plates are also used to restrain the AAC strip panels surrounding the opening. After the strip panels are assembled, the specified mortar is utilized to fill the gaps between the strip panels. The dimensions of the Type a and Type b wall units are 3000 mm × 2880 mm and 3012 mm × 2880 mm, respectively. The structural design diagrams of two types of wall panels are shown in Figures 1(a) and 1(b). The physical diagrams of the two types of wall panels are shown in Figures 2(a) and 2(b).

To complete the loading pushover test of the wall panel with and without windows, the parameters of the embedded ALC integrated wall panel are modified slightly. Two frame beams and two wall panels are adopted for the experiment. The overall wall panel size is 3050 × 2880 × 200 mm, the cross-section size of the frame beam is HM 200 × 200 × 8 × 12 mm, and the wall panel is classified into two kinds: with and without windows. The subsequent tests are performed on this wall panel.

### 2.3. Loading Test

#### 2.3.1. Loading System

**Figure 3:**
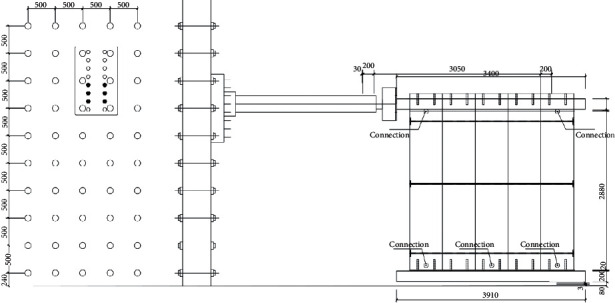
The structure diagram of the loading system.

The related experiments in this study are all carried out in the Disaster Prevention and Mitigation Structure Laboratory of Nanjing Tech University, and the pseudo-static loading system of MTS Corporation is adopted for controlling loading [20]. The structure diagram of the loading system is shown in Figure 3.

#### 2.3.2. Loading Setting

**Figure 4:**
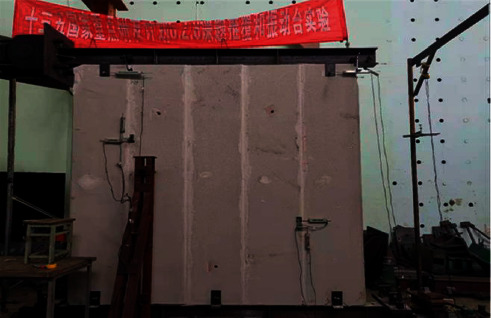
Schematic diagram for the operation of the ALC wall panel test.

The designed ALC wall panels apply low-cycle reciprocating horizontal loading to the system panels. Because of the brittleness of AAC slabs, the displacement-controlled hierarchical loading technology has been adopted [21]. The specific loading process during the test is shown in Figure 4.

**Figure 5:**
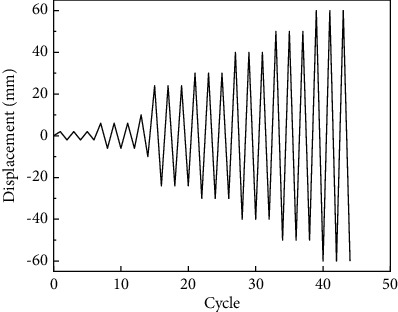
Displacement loading procedure.

Before the formal loading, a preloading with a small imposed displacement is performed to check whether the test piece is installed in place and whether the system work normally. Firstly, the load is applied to the imposed displacement of 6 mm, taking 2 mm as the first-level interval, and each load is performed for 1 cycle. Then, 1/250, 1/150, 1/50, and other important interlayer displacement angle limits were undertaken as control factors of displacement loading, and each level of loading is cycled 3 times. The loading procedure is shown in Figure 5.

### 2.4. Design of ALC Wall Panel Connection Joints

In this experiment, the pretightening force between the ALC wall panel assembly unit body and the connecting panel (the vertical part of the L-shaped connecting panel) directly determines the magnitude of the frictional force between the wall panel unit and the connecting pieces in the vibration direction, so the performance test is vital. The theoretical analysis shows that when the pretightening force of the connecting bolts is too small, the friction between the ALC panels assembly unit body and the connecting panel is also not large and relative sliding may occur in the case of small vibration, so that the contribution of ALC wall panels to the overall structural rigidity and bearing capacity will not be reflected. The pretightening force of the connecting bolt has the same change as the friction between the ALC panel assembly unit body and the connecting panel. The latter may cause “locking” between the ALC wall panels but no relative sliding of the overall structure in the case of strong vibration. In this case, it will generate large interlayer deformation of the ALC panel assembly unit body and cause damage to the wall itself, such as cracking. These all fail to meet the functional requirements of the building. Therefore, it is extremely important to design a suitable bolt pretightening force, aiming to make the ALC panel assembly unit body and the structure joint, which can provide a certain rigidity and capacity bearing during small vibrations, so as to achieve “no slipping under weak vibrations, possible slipping under moderate vibrations, and slipping under strong vibrations.”

Perforated bolts (rods) are adopted to connect the U-shaped holes on the connectors at each connection point in the ALC panel assembly unit body, and some bolt pretightening force is applied to further optimize the performance of ALC wall panels. Bolt pretightening force refers to the pretightening force in the direction of the bolt axis line generated between the bolt and the connected part under the action of the tightening torque in the process of screwing the bolt [22]. For a particular bolt, the magnitude of the pretightening force is related to the tightening torque of the bolt, the friction between the bolt and the nut, and the friction between the nut and the connected part. The pretightening effect can improve the reliability of the bolt connection, the antiloosening ability, and the fatigue strength of the bolt and enhance the tightness and rigidity of the connection.

The basic principles to design the bolt pretightening force in this experiment mainly include the following two points: no local pressure failure of ALC under the bolt end and no yield or break in the screw and hook. Due to the low strength of the ALC material (4 MPa), attention should be paid to the local pressure failure of the ALC material under the bolt hook during the bolt pretightening process. In this test, the local compressive stress is set to 3 times the compressive strength of the ALC material according to the local compressive characteristics of the ALC material under the bolt hook head.

The design value of bolt pretightening force *F*_0_ is as follows:

(1) $$\begin{array}{c} F_{0}=\left(0\cdot 6∼0\cdot 7\right)f_{y}\cdot A,\\ F_{0}=\left(0\cdot 6∼0\cdot 7\right)\sigma_{c}\cdot A_{\text{c}}. \end{array}$$

The bolt pretightening force is the smaller value of 60%∼70% of the yield force of the bolt rod and the local pressure bearing capacity. The relationship between bolt pretightening force and torque (tightening torque) is as follows:

(2) $$\begin{array}{c} T=K\cdot F\cdot d. \end{array}$$

The pretightening torque is 30∼42 Nm, which is calculated based on the relevant parameters in the experiment, and the corresponding screw pretightening force is 21.4∼30 kN. The pretightening torque is set as 40 Nm in this test.

### 2.5. Theoretical Analysis of Test Parameters

The mechanical characteristics of ALC wall panels are explored in this study, including the hysteresis curve, skeleton curve, and stability degradation analysis.

The hysteresis curve refers to the load-displacement curve obtained by the structure or component under the cyclic load. The shape of the hysteresis curve is a comprehensive manifestation of the seismic performance of the joints [23], and it can reflect the characteristics of stiffness degradation, strength attenuation, ductility performance, and energy consumption capability of the joints. In actual operation, the load-displacement curve of a structure or component under reciprocating loads is referred to as the hysteresis curve, which is the basis for analyzing the hysteresis performance of the structure. Analysis on the hysteresis curve can fully reflect the seismic performance, energy consumption, and structural ductility [24]. Because the calculated bearing capacity of the connection between the ALC outer wall panel and the I-beam is relatively small, displacement control is adopted when the reciprocating load is applied to the development connection. The displacement controls of different modulus are adopted based on various connected parameters [25].

The skeleton curve mainly refers to the outer envelope line connected by the peak points of the first cycle of loading at all levels in the load-displacement curve under the action of complex loads [26]. The skeleton curve is very similar to the load-displacement curve when the specimen is monotonously loaded, and it can also qualitatively measure the seismic performance of the structure. The curve is a very important parameter in the study of inelastic seismic response. It is the trajectory of the maximum peak point of each level of the load-displacement curve. During any loading process, the peak point cannot go beyond the skeleton curve but can only move forward along it [27]. Characteristics of the component such as the yield load and displacement, ultimate load, and displacement can be displayed very prominently on the skeleton curve. Besides, the curve can reflect the mechanical properties of the structure or components such as energy absorption and dissipation, ductility, strength, and stiffness degradation under the action of cyclic loading [28].

The skeleton curve is composed of the peak points of each level of the load-displacement curve. It can reflect the strength and ductility characteristics of the structure during the entire loading process, but it cannot reflect the stiffness characteristics of the structure under a specific loading displacement value [29]. The change of joint stiffness is an important parameter to evaluate the performance of connection joints, which can reflect the seismic performance of the joints [30]. Equivalent stiffness is adopted to evaluate the stiffness characteristics of the specimen in this study. Equivalent stiffness is also called secant stiffness, which is the slope of the straight line connecting the maximum load in the forward and reverse directions of each load. Its expression is given as follows:

(3) $$\begin{array}{c} K_{i}=\left(\frac{\left|+p_{i}\right|+\left|-p_{i}\right|}{\left|+\Delta_{i}\right|+\left|-\Delta_{i}\right|}\right). \end{array}$$

Here, +*p*_*i*_ and −*p*_*i*_ are the positive and negative peak loads of the *i*th level loading cycle and +△_*i*_ and −△_*i*_ are the positive and negative displacements of the *i*th level loading cycle.

### 2.6. Data Acquisition and Analysis of the Experimental Instruments

In this experiment, the MS COCO dataset, which is a large-scale dataset for object detection, segmentation, key-point detection, and captioning, is applied in the lightweight deep learning target detection algorithm. This dataset consists of 328,000 images, and the official website is https://cocodataset.org/#home.

According to the specific experimental parameters involved in the wall panel experiment and its own experimental conditions, the experimental data are obtained from the wall panel experiment to better analyze and observe the experimental phenomena and experimental results. The main collection methods are as follows.

Due to the low-cycle reciprocating horizontal load applied by MTS during the test, the system can automatically record the load applied by the hydraulic servo actuator and output it in the form of voltage [31]. The DH3826 dynamic strain collector is adopted to automatically collect and record the load parameters and draw the load-displacement hysteresis curve of the structure based on the load value. Because the displacement of the hydraulic servo actuator of MTS shows errors in the jack itself and the structure, the displacement value generated is generally not directly used in the test results. The external displacement value of the structure is collected as the standard value of the load-displacement hysteresis curve. The external displacement of the structure is measured using a displacement meter. The output voltage signal of the displacement meter is automatically recorded using the DH3826 dynamic strain acquisition instrument, and the sampling frequency is 10 Hz. The crack development of the ALC wall panel is observed during the test and related test phenomena are recorded. The damages of the wall panel joints and connection joints are observed and recorded with a marker, so as to determine the crack failure form of the wall panel.

## 3. Experimental Observation

### 3.1. Test Phenomenon of the Wall Panel with Windows

**Figure 6:**
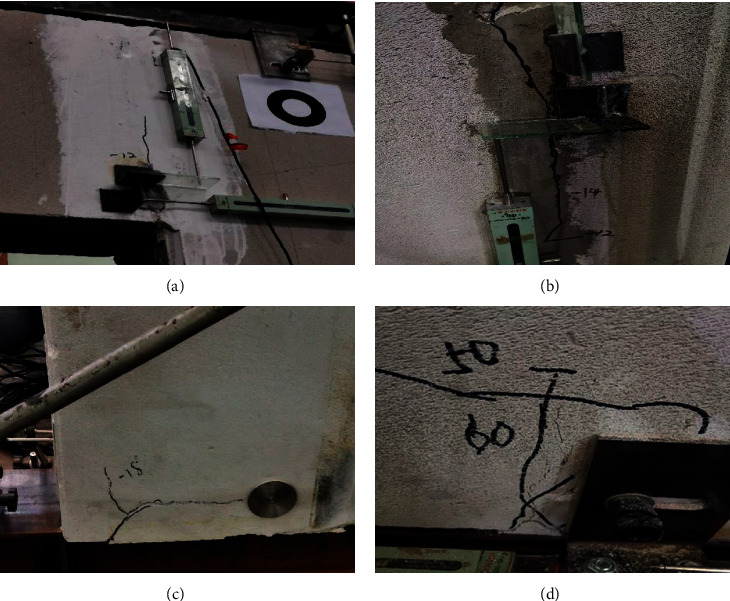
Destruction process and destruction form of the panel with windows. (a) Cracks in the wall panel. (b) Crack extended. (c) Cracks in the corner. (d) Crack at the joint extended.

At the initial stage of loading, the structure is in the elastic stage. When the imposed displacement is increased to 12 mm, the vertical cracks begin to appear between the joints of the wall panel, as shown in Figure 6(a). When the imposed displacement is 14 mm, the cracks begin to extend, as shown in Figure 6(b), and the joint bolts were observed to slip, resulting in relative slippage between the wall panel and loading beams. During the subsequent loading process, the cracks between the panels keep propagating and wide cracks appear at the corner, as shown in Figure 6(c). When the imposed displacement reaches 24 mm, the bearing capacity of the test ALC wall panel decreases greatly and the length of the cracks at the corner is significantly increased. When it comes to the later stage of loading, the cracks in the concrete near the joint extend and the crack width also increases significantly. The failure process and failure mode of the wall panel with windows are shown in Figure 6.

### 3.2. Test Phenomenon of the Wall Panel without Windows

**Figure 7:**
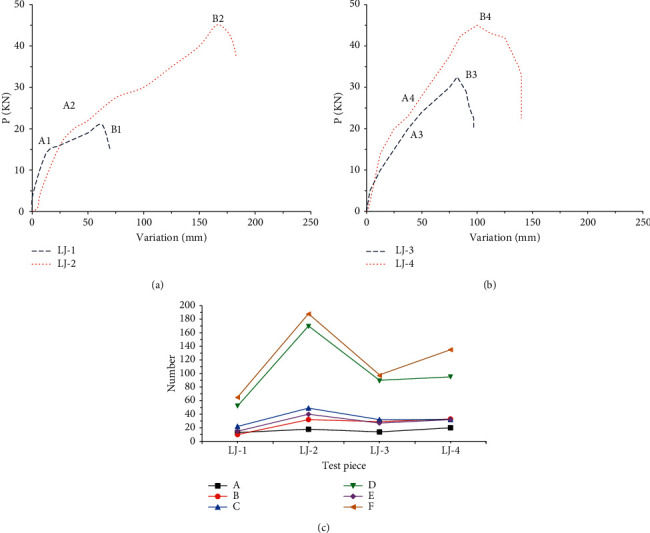
Load-displacement curves under monotonic loading (A: yield load; B: yield displacement; C: ultimate load; D: ultimate displacement; E: failure load; F: failure displacement). (a) Load-displacement curves of LJ-1 and LJ-2. (b) Load-displacement curves of LJ-3 and LJ-4. (c) Various indexes of LJ-1∼LJ-4.

**Figure 8:**
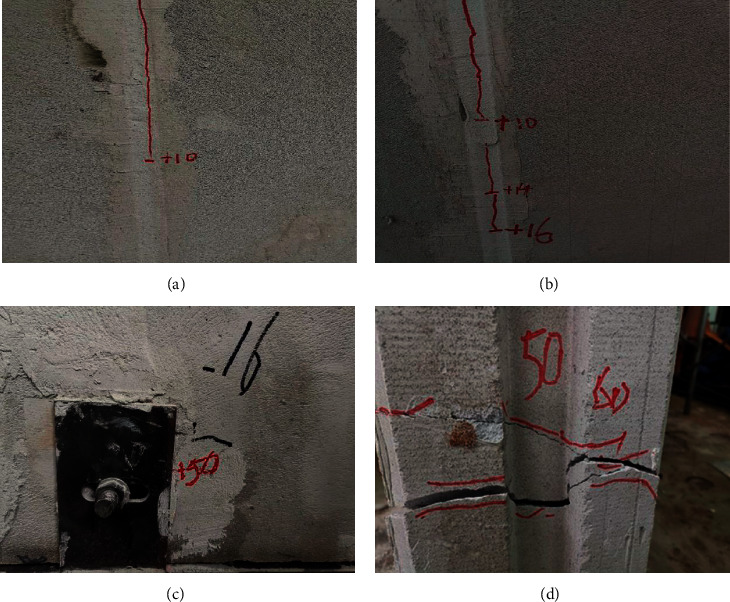
Destruction process and destruction form of the panel without windows. (a) Cracks in the wall panel. (b) The crack length extended. (c) Cracks in the corner. (d) Crack is widened.

The loading process and the observed phenomenon of the ALC wall panel without windows (Type a) are quite similar with those for the ALC wall panel with windows (Type b). Firstly, when the initial imposed loading displacement is 2 mm, there is no obvious damage on the surface of the wall panel. When the imposed displacement is up to 10 mm, fine cracks begin to gradually appear between the joints of the wall panel, as shown in Figure 7(a). When the imposed displacement is 14 mm, the cracks begin to extend, as shown in Figure 7(b), and the joint bolts were observed to slip, resulting in relative slippage between the wall panel and loading beams. When the imposed displacement reaches 16 mm, cracks appear near the joints, as shown in Figure 7(c). When the maximum imposed displacement is reached, part of the ALC material near the joint was crushed and the crack width is close to 1 mm, as shown in Figure 7(c). The failure process and failure mode of the wall panel without window opening are shown in Figure 8.

## 4. Experimental Results and Discussion

### 4.1. Performance of Connecting Nodes of Wall Panels under Monotonic Loads

Figure 7 shows the load-displacement curve under monotonic loading.

Based on Figures 7(a) and 7(b), the cracking processes of the nodes under monotonic load are described. The destruction process of LJ-1 is as follows: when the vertical load is loaded to 11 kN, the carbon fiber cloth makes noise. At this point, the wall panel has been damaged and the main force is the carbon fiber cloth and steel bars in the wall panel. As the load continues to increase, the displacement continues to increase and the carbon fiber cloth begins to tear and fail. When the load is loaded to 22 kN, two vertical cracks are torn on the front of the carbon fiber cloth and the ALC wall panel shows debris falling off, the steel bars yield could no longer bear the force and the connecting bolts are slightly bent. The destruction process of LJ-2 is as follows: when the vertical load is loaded to 20 kN, the carbon fiber cloth makes noise and the load continues to increase. When the load is loaded to 40 kN, a vertical crack is torn on the front of the carbon fiber cloth, the ALC wall panel has debris falling off, and the connecting bolts are seriously yielded and cannot continue to bear the force. The destruction process of LJ-3 is as follows: when the vertical load is loaded to 20 kN, the carbon fiber cloth makes noise. As the load continues to increase, the displacement continues to increase and the carbon fiber cloth begins to tear and damage. When the load reached 29 kN, two vertical cracks are torn on the front of the carbon fiber cloth, the yield deformation of the screw is obvious, and the short angle steel of the connecting piece is slightly deformed, so it could not continue to bear the force. The destruction process of LJ-4 is as follows: when the vertical load is loaded to 20 kN, the carbon fiber cloth makes noise. As the load continued to increase, the carbon fiber cloth began to tear and damage. When the load is loaded to 40 kN, a vertical crack is torn on the front of the carbon fiber cloth, the ALC wall panel has debris falling off, and the connecting bolts are broken, which cannot continue to bear the force. The bearing capacity curves of the four nodes show the same trend; that is, they first rise and then fall until the end of the load.

As shown in Figure 7(a), for the connection of LJ-1, the yield displacement is about 10 mm and the connecting screw does not yield significantly. For the connection of LJ-2, not only are the wall panel and carbon fiber cloth badly damaged but the connecting screws are cut and no more force could be applied. When the ultimate bearing capacities of LJ-1 and LJ-2 are 21.3 kN and 46.17 kN, the corresponding displacements are 54.9 mm and 166.37 mm, respectively. This shows that the two connection forms clearly meet the specification requirements in terms of deformation and force. The comparative analysis of the two connections under monotonic load shows that the ultimate bearing capacity of the joint on the development connection is about 46 kN under monotonic load. The displacement of LJ-2 at failure is much larger than that of LJ-1, indicating that the synergistic working ability of 200 mm thick wall panel and carbon fiber cloth is much better than that of 150 mm thick wall panel and the bearing capacity of LJ-2 is significantly higher than that of LJ-1. The seams meet specification requirements. Figure 7(b) shows that the ultimate load of LJ-3 is 29.32 kN and the ultimate displacement is 88.81 mm. The ultimate load of LJ-4 is 40.5 kN, and the ultimate displacement is 92.08 mm. The connecting bolt of LJ-4 has been cut off by the final failure. Figure 7(c) shows that the ultimate bearing capacity deformation of the joint under the developing connection is about 41 kN, while the displacement of LJ-4 at fracture is slightly higher than that of LJ-3. However, in the A4B4 section, the rising speed of LJ-4 is significantly accelerated, indicating that the 200 mm plate thickness and carbon fiber have strong synergistic working ability and the ultimate bearing capacity of LJ-4 is much lower than that of LJ-3 in the A4B4 section. However, the displacement is not much different at this time. When the thickness of the wall panel is designed, only the bearing capacity can meet the requirements of this area and the joint of the thickness of the two panels meets the requirements of the specification.

### 4.2. Analysis on the Hysteresis Curve of Experiment Components

**Figure 9:**
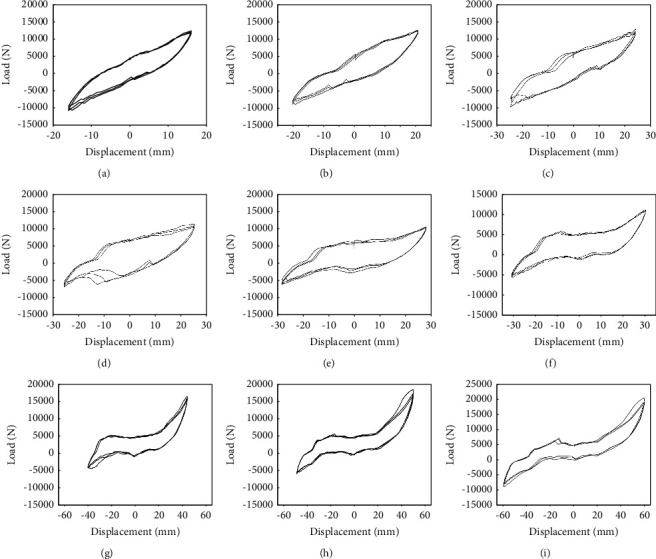
The hysteresis curves of ALC wall panel with windows. (a) Displacement value = 16 mm. (b) Displacement value = 20 mm. (c) Displacement value = 24 mm. (d) Displacement value = 26 mm. (e) Displacement value = 28 mm. (f) Displacement value = 30 mm. (g) Displacement value = 40 mm. (h) Displacement value = 50 mm. (i) Displacement value = 60 mm.

The data acquisition system that comes with MTS is adopted to obtain the load-displacement curve of the structure. The imposed displacement values for ALC wall panels with windows are 16 mm, 20 mm, 24 mm, 26 mm, 28 mm, 30 mm, 40 mm, 50 mm, and 60 mm, respectively, and the specific results are shown in Figure 9. The imposed displacement values for the ALC wall panels without windows are 16 mm, 18 mm, 20 mm, 24 mm, 28 mm, 32 mm, 40 mm, 50 mm, and 60 mm, respectively. The specific results are shown in Figure 9.

The vertical data in Figure 9 refer to the load capacity. Figure 9 illustrates that in the ALC wall panels with windows, when the loading displacement is 16 mm, 20 mm, and 24 mm, the areas enclosed by the hysteresis curve all present a fusiform as a whole and the curve basically shows a linear change. It shows that when the loading displacement is small, the embedded screw is firmly connected to the wall panel, the displacement change is tiny, and the bearing capacity increases faster. With the gradual increase of loading displacements, the hysteresis curve is no longer linear. At this stage, the curve displacement changes quickly and the bearing capacity changes slowly. Then, the force and displacement gradually approach the linear changes, indicating that the screw and wall panel are connected firmly. At this time, there are a few cracks on the wall panel surface near the embedded screw. It is found that during the loading, there is an obvious pinching in the middle of the hysteresis loop, which indicates that there is a certain shear slippage in the development connection. This slippage mainly occurs between the connecting angle and the wall panel and between the screw and the connecting angles. Slipping slows down the damage of the wall panel and also helps to improve the seismic performance of the wall panel structure.

**Figure 10:**
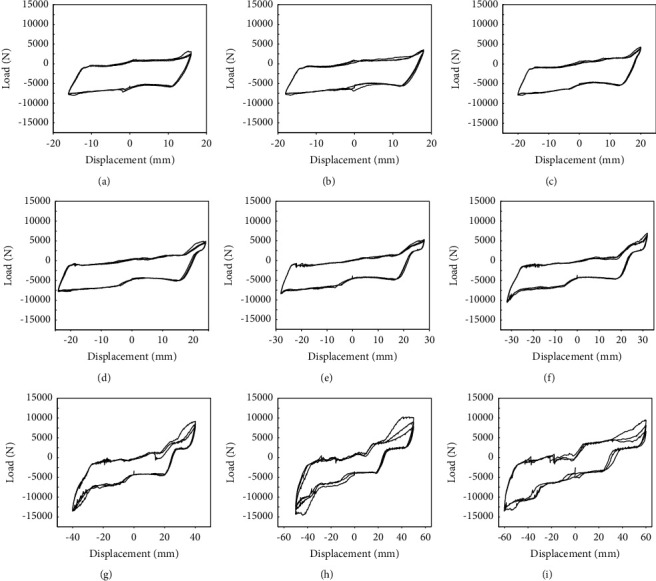
The hysteresis curves of the ALC wall panel without windows. (a) Displacement value = 16 mm. (b) Displacement value = 18 mm. (c) Displacement value = 20 mm. (d) Displacement value = 24 mm. (e) Displacement value = 28 mm. (f) Displacement value = 32 mm. (g) Displacement value = 40 mm. (h) Displacement value = 50 mm. (i) Displacement value = 60 mm.

The vertical data in Figure 10 refer to the load capacity. The pinching is also found in the hysteresis curve of ALC wall panels without windows, and the pinching becomes more and more obvious with the increase in the loading degree. At the beginning of loading, the enclosed area of the hysteresis curve of ALC wall panels is tiny. As the loading displacements increase, the area enclosed by the hysteresis curve begins to increase. From the overall perspective of the hysteresis curve, the curves of pressure and tension applied by connection joints are asymmetric. During the whole loading process, the rigidity of the structure gradually decreases with the increase in displacement and stiffness degradation appears. At this time, the connecting screw also has different degrees of deformation.

### 4.3. Skeleton Curve

**Figure 11:**
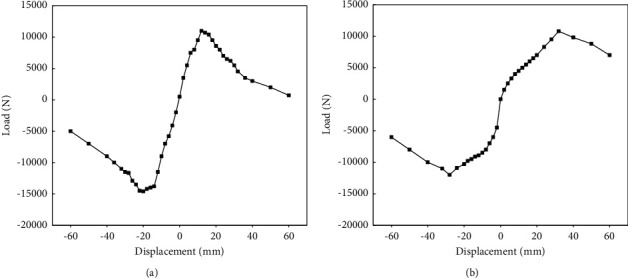
Skeleton curves of ALC wall panels. (a) ALC wall panel with windows. (b) ALC wall panel without windows.

In addition to the above analysis, the changes of the skeleton curve for ALC wall panels without and with windows are explored and analyzed, and the specific results are shown in Figures 11(a) and 11(b).

Figure 11 discloses that the skeleton curve of ALC wall panels with and without windows all basically shows an “*S*”-shaped trend and the degree of stiffness degradation is obvious. However, with the increase in displacement, the skeleton curve under the condition of without windows is smoother than that under the condition of with windows, indicating that the ALC wall panels without windows are more restrictive to the connecting screw and the co-working effect between the wall panel and connecting screw is more obvious. There is a downward trend in the skeleton curve of ALC wall panels under both conditions (without and with windows), which may be caused by the pretightening force of the bolts between the ALC wall panels and the bolts. It delays the damage of the connection and shows slow attenuation.

### 4.4. Stiffness Degradation Analysis

**Figure 12:**
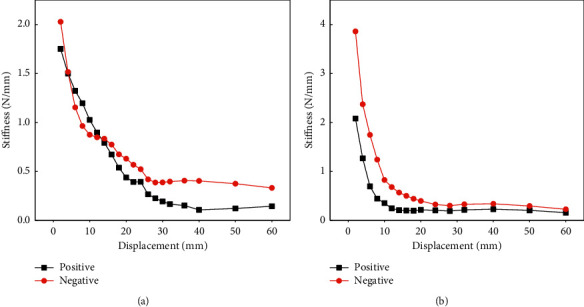
Stiffness degradation curves of ALC wall panels. (a) ALC wall panel with windows. (b) ALC wall panel without windows.

Comparison on stiffness degradation curves of ALC wall panels under the conditions of with and without windows (as illustrated in Figures 12(a) and 12(b)) shows that the positive initial stiffness of ALC wall panels with windows is 1.75 N/mm and the negative initial stiffness is 2.028 N/mm, while the positive and negative initial stiffness of the ALC wall panels without windows is 2.08 N/mm and 3.86 N/mm, respectively. In addition, it is found that the stiffness curve of ALC wall panels without windows drops obviously faster, which also shows that the good connection between the wall panel and the steel beam causes serious damage to the wall panel. However, there is a flat level when the displacement is 20 mm–60 mm, indicating that the stiffness degradation has been effectively alleviated.

## 5. Discussion

This work focuses on the performance of ALC wall panels. Under the condition of windows, when the test load-displacement is small, the area enclosed by the hysteresis curve is shuttle-shaped as a whole and changes linearly and the bearing capacity changes slowly with the increase in the load displacement. The structural stiffness of ALC wall panels degrades in windowless condition. There is clear clamping in the middle of the hysteresis curves of the two types of ALC wall panels, which indicates shear slip at the joints of the wall panels. The sliding slows down the damage of the wall panel, which is beneficial to improving the seismic performance of the wall panel structure. Under the condition of windows and no windows, the skeleton curve of the ALC wall panel basically shows an “*S*”- shaped trend. Since the ALC wall panels participated in the frame energy dissipation and earthquake resistance during the experiment, the skeleton curve of the windowless ALC wall panels is smoother, which indicates that the wall panels have greater restrictions on connecting screws. Bolt preload between ALC siding and bolts can delay joint damage. The stiffness degradation analysis shows that the positive initial stiffness of the windowed and nonwindowed ALC wall panels are 1.75 N/mm and 2.08 N/mm, and the negative initial stiffness is 2.028 N/mm and 3.86 N/mm, respectively. The decline rate of the stiffness curve of the windowless ALC wall panel is significantly faster. Therefore, good connection between the wall panel and the steel beam has caused serious damage to the wall panel.

## 6. Conclusions

Cyclic tests are performed on two types of ALC wall panels (with and without windows) based on a quasi-static loading system. In addition, the performance of the wall panels and the performance of the lightweight deep learning algorithm are discussed and analyzed and the seismic performance of the ALC wall panels is analyzed in combination with the preload study. In this work, the lightweight deep learning algorithm is applied to the research of ALC wall panels, which provides a reference for the further research and development of deep learning technology in the field of building earthquake resistance. In addition, the performance of ALC wall panels with and without windows is analyzed from multiple perspectives, and the superiority of the connection function of the wall panels is highlighted, providing scientific and effective reference materials for the subsequent analysis of the seismic effect of ALC wall panels. According to the actual engineering practice and the basic situation of actual ALC wall panel engineering application, the ALC wall panels with and without windows are innovatively analyzed. The research results are helpful in exploring the practical application effect of embedded ALC wall panels, showing important practical application value. There are still some deficiencies in this work. Because there are few studies on the seismic performance of prefabricated wall panels, the comparison of experimental conclusions is lacking in this paper, which makes the conclusions of the paper less convincing. The performance of the lightweight deep learning algorithm has not been compared and tested, and the degree of integration with the performance research of wall panels is not enough. The experimental research in this work still has a certain gap with the seismic performance under earthquake action in engineering practice and cannot truly reflect the seismic resistance of the structure under actual earthquake action. Therefore, in the follow-up research, it will carry out more in-depth research on these three aspects to obtain more realistic and convincing conclusions.

## Data Availability

The data used to support the findings of this study are included within the article.
